# Relationship Quality Predicts Advance Care Planning Engagement In Families Experiencing Cognitive Decline

**DOI:** 10.1093/geroni/igab046.3430

**Published:** 2021-12-17

**Authors:** Rachel Bloom, Celia Fisher

**Affiliations:** 1 Fordham University, New York, New York, United States; 2 Fordham University, Bronx, New York, United States

## Abstract

Although clinical guidelines recommend that people showing signs of cognitive decline engage in Advance Care Planning (ACP) while they still have decision-making capacity, too often this opportunity to clarify values and treatment preferences is missed among patients and their families. In reflection of the paucity of empirical data on factors influencing how this planning process begins for families experiencing cognitive decline, this study explored facilitators and barriers to ACP among adult children of parents showing signs of early- to mid-stage dementia, with a particular focus on relationship quality. Among this sample (N = 315), relationship quality positively and significantly predicted advance care planning engagement (r = .349, p < .001). Financial burden weakly and positively predicted ACP engagement (r = .123, p <.05), while both psychological burden (-.614, p < .001) and financial burden (-.290, p < .001) negatively and significantly predicted relationship quality. This study validates the use of the ACP Engagement Survey (ACPES) adapted for surrogates among adult children of people experiencing cognitive decline and contributes to a scarce literature on the impact of relationship quality on ACP engagement.

